# Supplementary material for: Predicting visual function by interpreting a neuronal wiring diagram
Source: Nature. 2024 Oct 2;634(8032):113–23. doi: 10.1038/s41586-024-07953-5 (PMC11446822; doi:10.1038/s41586-024-07953-5)
Supplement: Supplementary file 6 — CRF and ERF predictions for individual TmY4 and TmY9 cells. Analogous to Supplementary Data 3, but for TmY target types. Shown are the top four monosynaptic pathways, the strongest pathway passing through each of the top ten intermediary types (ranking from Extended Data Fig. 7), and the trisynaptic pathway Tm1–TmY–Dm3–TmY (see the section entitled Prediction of spatial normalization). [file 41586_2024_7953_MOESM6_ESM.zip › DataS4/TmY9qΓèÑ/720575940605937766.pdf]

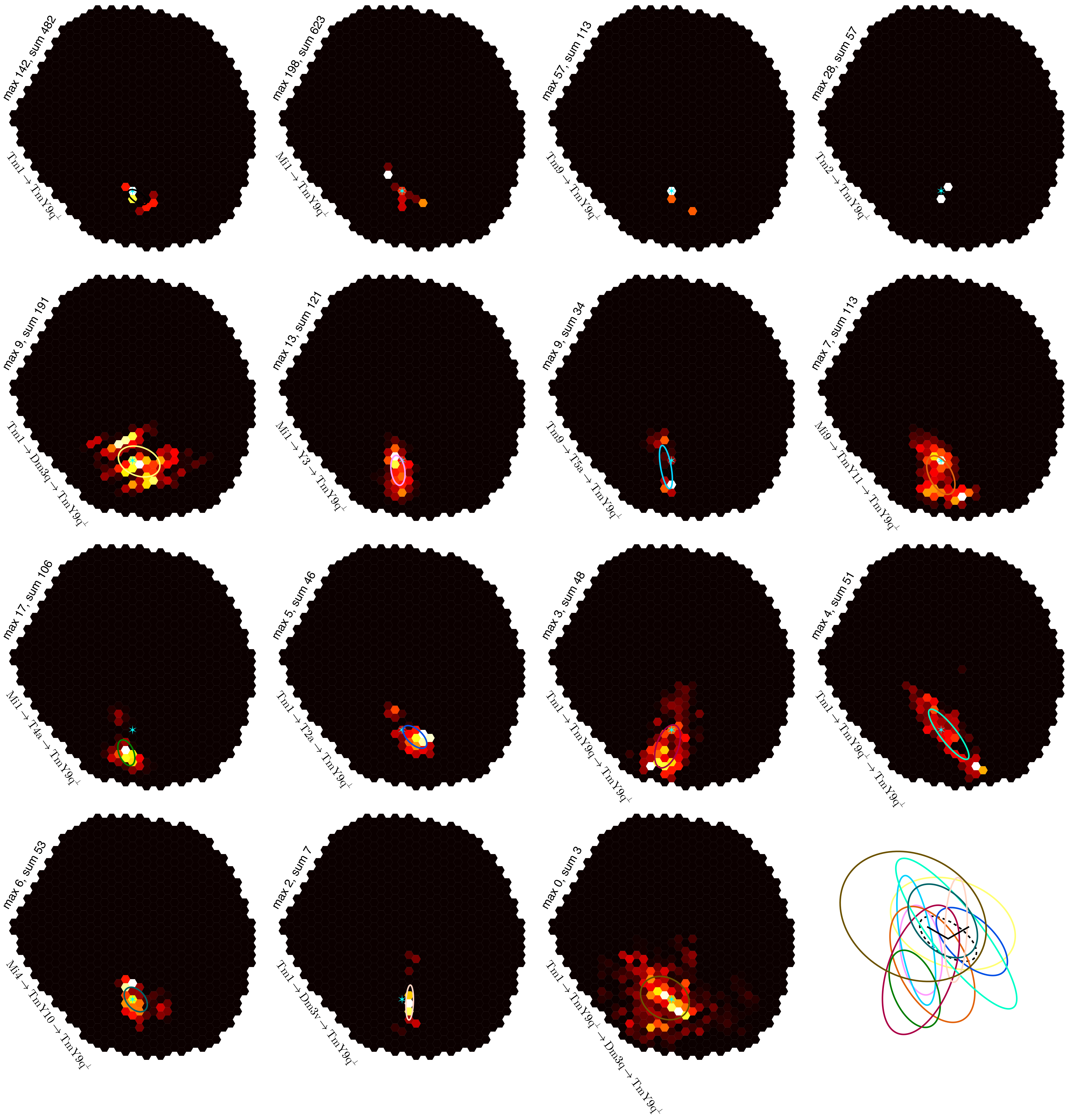
max 142, sum 482
Tm1 → TmY9q⊥
max 198, sum 623
Mi1 → TmY9q⊥
max 57, sum 113
Tm9 → TmY9q⊥
max 28, sum 57
Tm2 → TmY9q⊥
max 9, sum 191
Tm1 → Dm3q → TmY9q⊥
max 13, sum 121
Mi1 → Y3 → TmY9q⊥
max 9, sum 34
Tm9 → T5a → TmY9q⊥
max 7, sum 113
Mi9 → TmY11 → TmY9q⊥
max 17, sum 106
Mi1 → T4a → TmY9q⊥
max 5, sum 46
Tm1 → T2a → TmY9q⊥
max 3, sum 48
Tm1 → TmY9q → TmY9q⊥
max 4, sum 51
Tm1 → TmY9q⊥ → TmY9q⊥
max 6, sum 53
Mi4 → TmY10 → TmY9q⊥
max 2, sum 7
Tm1 → Dm3v → TmY9q⊥
max 0, sum 3
Tm1 → TmY9q⊥ → Dm3q → TmY9q⊥
